# Physical Activity and Perceptions of the Community Walking Environment Among Cancer Survivors, National Health Interview Survey, 2024

**DOI:** 10.5888/pcd23.260220

**Published:** 2026-10-22

**Authors:** Jonathan Bryant-Genevier, Michael Boring, Elizabeth A. Rohan, Dawn M. Holman, Anne K. Julian, Yamisha Bermudez, Mary Puckett, Shixiu Ricardo, Jasmine Y. Nakayama

**Affiliations:** 1 Division of Cancer Prevention and Control, National Center for Chronic Disease Prevention and Health Promotion, Centers for Disease Control and Prevention, Atlanta, Georgia; 2 Division of Nutrition, Physical Activity, and Obesity, National Center for Chronic Disease Prevention and Health Promotion, Centers for Disease Control and Prevention, Atlanta, Georgia

## Abstract

**Introduction:**

Cancer survivors are a growing population in the US. Physical activity can reduce cancer-specific and all-cause mortality and decrease cancer recurrence risk among survivors. However, estimates of physical activity among cancer survivors by sociodemographic and health factors are not up to date.

**Methods:**

We used data from the 2024 National Health Interview Survey of civilian, noninstitutionalized US adults to calculate the proportions of cancer survivors who were inactive, insufficiently active, and sufficiently active through meeting leisure-time aerobic physical activity guidelines (0, 1–149, and ≥150 minutes/week of moderate-intensity equivalent physical activity, respectively) by sociodemographic and health factors and examined associations between physical activity and perceptions of the local walking environment.

**Results:**

In 2024, based on our sample (N = 3,486), an estimated 21.1 million US adults reported a history of cancer. Among cancer survivors, 33.9% (95% CI, 31.9%–35.9%) were inactive, 28.1% (95% CI, 26.3%–30.1%) were insufficiently active, and 38.0% (95% CI, 36.0%–40.0%) were sufficiently active. Cancer survivors living in communities with features that support walking reported lower levels of inactivity compared with survivors living in communities without such supports. Physical inactivity was higher among those with multiple comorbid chronic conditions and those reporting fair or poor overall health.

**Conclusion:**

Levels of physical activity among cancer survivors are low, especially for survivors with additional comorbid chronic conditions or who report fair or poor overall health. Evidence-based physical activity interventions that address land use and environmental design alongside community engagement to promote walking may increase physical activity among cancer survivors.

**Summary**

**What is already known on this topic?**

Physical activity can reduce cancer-specific mortality, all-cause mortality, and recurrence risk among cancer survivors. The built environment can influence the convenience and feasibility of integrating physical activity into daily activities.

**What is added by this report?**

The prevalence of physical inactivity among cancer survivors in the US was higher among those with multiple comorbid chronic conditions, those reporting fair or poor overall health, and those living in communities without features that support walking.

**What are the implications for public health practice?**

Built environment features may increase aerobic physical activity among cancer survivors. Public health practitioners and interdisciplinary partners may consider evidence-based strategies incorporating land use, environmental design, and community engagement to enhance aerobic physical activity opportunities.

## Introduction

Cancer survivorship is an expanding public health concern. The number of people alive in the US who have ever been diagnosed with cancer (cancer survivors) is projected to increase from 18 million in 2022 to 26 million by 2040 (1–3). This growth reflects an aging population and improvements in cancer prognosis driven by increased screening, earlier detection, and advances in treatment (4,5). Nearly half of survivors have lived 10 years or more since diagnosis, and almost 1 in 5 have lived 20 years or more (1). An increasing proportion of survivors also live with multiple chronic comorbid conditions, which complicate clinical management, increases burdens on care teams and caregivers, and are associated with more days of poor mental and physical health and elevated mortality risk (6–8).

Regular physical activity can provide numerous health benefits for cancer survivors, including reduced cancer-specific and all-cause mortality, lower risk of recurrence, fewer comorbidities, mitigation of treatment-related side effects, and improved emotional and psychosocial health (9–12). Increasing physical activity among adults with low physical activity has substantial health benefits (13). In addition, most comprehensive cancer control plans incorporate some planning to improve physical activity in their populations, indicating a desire to support survivors in this area (14). However, many cancer survivors do not achieve recommended activity levels. In 2020, 55% of cancer survivors reported (9) not meeting the federal aerobic physical activity guidelines described in Physical Activity Guidelines for Americans (10,12) (ie, ≥150 minutes/week of moderate-intensity activity, 75 minutes/week of vigorous-intensity activity, or an equivalent combination of moderate- and vigorous-intensity activity) (9). The American Cancer Society Physical Activity Guideline for Cancer Survivors recommends the same levels of physical activity and additionally recommends that physical activity assessment and counseling begin as soon as possible after a cancer diagnosis and that survivors consult their treatment team before starting or changing their physical activity plan (10).

Community environments can be designed to promote many forms of aerobic physical activity, including running, biking, and walking, which is a common and accessible form of physical activity among people with disabilities who walk or use assistive devices (15–22). For example, adults living in more walkable neighborhoods are more likely to engage in walking (20,23). Built environment features, such as proximity to everyday destinations, land-use patterns, and access to public transit, influence the convenience and feasibility of integrating walking and other forms of physical activity into daily activities.

Previous research has studied self-reported and accelerometer-measured physical activity, including walking behaviors, among cancer survivors (22,24–26). However, estimates of the prevalence of physical activity and built environment features among all cancer survivors are not current (9,27). We analyzed data from a sample of US cancer survivors from nationally representative data to update estimates of the prevalence of leisure-time aerobic physical activity levels among cancer survivors by sociodemographic and health factors. We also examined associations with features of the local community walking environment individually.

## Methods

We used adult interview data from the 2024 National Health Interview Survey (NHIS). The NHIS is a continuous cross-sectional household survey designed to generate a nationally representative sample of the noninstitutionalized US civilian population and is described elsewhere (28). We measured self-reported history of cancer (ie, cancer survivor) as a response of yes to the question, “Have you ever been told by a doctor or other health professional that you had cancer or a malignancy of any kind?” This survey question identified 4,434 cancer survivors among 32,629 respondents. We excluded respondents if their only reported cancer diagnosis was nonmelanoma skin cancer or unknown skin cancer (n = 948), resulting in an analytic sample of 3,486 respondents. As part of NHIS interviews, respondents self-reported their age, sex, race and ethnicity, education level, marital status, height and weight (used to determine body mass index), health status, smoking status, and disability status (29). We used the 2023 National Center for Health Statistics Urban–Rural Classification Scheme for Counties to categorize respondents’ county of residence according to urbanization levels (29). The survey also collects data on the number of chronic conditions reported by respondents. These were measured according to affirmative responses to the following conditions: arthritis, chronic obstructive pulmonary disease, cirrhosis or hepatitis, dementia, diabetes, heart disease, hypertension, stroke, and weak or failing kidneys.

Leisure-time physical activity levels were assigned according to aerobic physical activity guidelines as inactive (0 minutes/week of activity), insufficiently active (1–149 minutes/week), or sufficiently active (≥150 minutes/week, ie, meeting federal guidelines) using self-reported frequency and duration of moderate-intensity equivalent aerobic activity (in which 1 minute of vigorous-intensity activity is equivalent to 2 minutes of moderate-intensity activity) (12). The NHIS assesses perceptions of the local walking environment by determining the presence of 9 features (6 supports and 3 barriers) of the environment, asking participants, “Where you live, are there . . . that you can walk to?” The 6 supports are 1) roads, sidewalks, paths, or trails (walking routes), 2) shops, stores, or markets (shopping), 3) bus or transit stops (transit), 4) places that help you relax, clear your mind, and reduce stress (relaxing), 5) places like movies, libraries, or places of worship (other), and 6) sidewalks. The 3 barriers were assessed similarly by asking participants, “Where you live, does . . . make it unsafe for you to walk?” These were traffic, crime, and dogs or other animals (animals). To assess weather-related barriers to walking, a Likert-type item asked, “How often does the weather make you less likely to walk?” Possible responses were almost always, most of the time, some of the time, and never.

We used the *survey* package in R version 4.5.0 (R Foundation for Statistical Computing) for all analyses (30,31). We stratified sample characteristics by sex due to differences in cancer type and prevalence by sex, although subsequent analyses of physical activity outcomes were unstratified (sex was included along with other characteristics as a variable). We calculated weighted prevalences and 95% CIs and estimated population counts, accounting for the complex survey design and adjusting for nonresponse (29). We used Rao–Scott χ^2^ tests to assess differences in physical activity levels among groups. To account for multiple comparisons, we conducted pairwise Bonferroni-corrected *t* tests separately for each physical activity level. All results were unadjusted, and significance was defined at *P* < .05. Estimates that did not meet reliability criteria of minimum effective sample size (≥30) and absolute and relative CI width were suppressed in accordance with National Center for Health Statistics data presentation standards for proportions (32).

## Results

In 2024, an estimated 21.1 million US adults (unweighted N = 3,486) reported a history of cancer other than nonmelanoma skin cancer or unknown skin cancer (55.4% female, 44.6% male) (Table 1). Of these, 40.0% were younger than 65 years (46.8% female, 31.5% male). Approximately one-third (34.0% overall, 35.2% female, 32.4% male) reported their health status as excellent or very good. Approximately one-quarter of cancer survivors (25.8% overall, 28.6% female, 22.4% male) reported no other chronic comorbid conditions beyond their cancer diagnosis, whereas nearly half (47.7% overall, 43.1% female, 53.3% male) reported 2 or more additional chronic conditions. An estimated 3 million cancer survivors reported a lot of difficulty walking or climbing steps or being unable to perform these activities at all (mobility disability) (14.3% overall, 15.2% female, 13.3% male).

**Table 1:** Characteristics of Self-Reported Cancer Survivors (N = 3,486), National Health Interview Survey, 2024^a^

Characteristic	Total			Male		Female	
	No.	Population (millions)	% (95% CI)	No.	% (95% CI)	No.	% (95% CI)
**Overall**	3,486	21.1	—	1,478	44.6 (42.5–46.7)	2,008	55.4 (53.3–57.5)
**Age group, y^b^**							
18–39	133	1.2	5.6 (4.4–6.7)	41	4.2 (2.8–5.5)	92	6.7 (5.1–8.3)
40–64	965	7.2	34.4 (32.4–36.4)	325	27.3 (24.3–30.2)	640	40.1 (37.3–42.8)
65–74	1,088	5.7	27.1 (25.5–28.8)	494	28.8 (26.1–31.4)	594	25.8 (23.6–28.0)
≥75	1,292	6.9	32.9 (31.1–34.8)	615	39.8 (37.0–42.7)	677	27.4 (25.1–29.7)
**Race and ethnicity**							
Hispanic	229	1.7	8.1 (6.8–9.4)	87	7.8 (5.8–9.7)	142	8.4 (6.7–10.0)
Non-Hispanic Black	279	2.0	9.3 (7.8–10.8)	123	9.5 (7.5–11.5)	156	9.1 (7.2–11.0)
Non-Hispanic White	2,803	16.2	76.5 (74.4–78.6)	1,211	78.2 (75.3–81.1)	1,592	75.2 (72.5–77.9)
Non-Hispanic Other	175	1.3	6.1 (4.9–7.2)	57	4.5 (3.0–6.1)	118	7.3 (5.6–9.0)
**Education level**							
Less than high school	277	2.0	9.3 (8.0–10.6)	121	10.5 (8.4–12.7)	156	8.4 (6.7–10.0)
High school or equivalent	893	5.5	26.1 (24.2–27.9)	372	26.9 (24.1–29.7)	521	25.4 (23.0–27.8)
Some college	984	5.9	28.2 (26.2–30.1)	375	24.4 (21.7–27.1)	609	31.2 (28.7–33.7)
College	1,325	7.7	36.4 (34.4–38.5)	607	38.1 (35.2–41.1)	718	35.0 (32.5–37.6)
**Marital status**							
Married/partnered	1,697	13.1	64.5 (62.6–66.5)	851	72.7 (70.1–75.3)	846	58.0 (55.3–60.7)
Widowed	707	2.9	14.0 (12.7–15.3)	188	8.8 (7.2–10.3)	519	18.2 (16.3–20.1)
Single/separated	963	4.4	21.5 (19.9–23.1)	382	18.5 (16.3–20.7)	581	23.8 (21.6–26.0)
**Urban–rural classification**							
Large central metropolitan	837	5.7	26.9 (24.4–29.5)	341	25.3 (21.9–28.7)	496	28.3 (25.2–31.3)
Large fringe metropolitan	711	5.3	24.9 (22.2–27.6)	325	26.0 (22.4–29.5)	386	24.0 (20.9–27.1)
Medium and small metropolitan	1,034	6.7	31.6 (28.3–34.8)	454	33.7 (29.5–37.9)	580	29.9 (26.5–33.3)
Rural	904	3.5	16.6 (14.9–18.3)	358	15.0 (12.8–17.3)	546	17.8 (15.7–20.0)
**BMI category**							
Underweight/healthy weight (<25 kg/m^2^)	1,129	6.7	32.4 (30.5–34.3)	427	27.5 (25.0–30.1)	702	36.4 (33.8–39.0)
Overweight (≥25 to <30 kg/m^2^)	1,184	7.2	34.8 (32.8–36.8)	621	42.5 (39.5–45.5)	563	28.5 (26.0–31.0)
Obesity (≥30 kg/m^2^)	1,115	6.8	32.8 (30.8–34.7)	424	30.0 (27.2–32.8)	691	35.1 (32.5–37.6)
**Health status**							
Excellent/very good	1,235	7.2	34.0 (32.1–35.9)	509	32.4 (29.7–35.2)	726	35.2 (32.5–37.8)
Good	1,205	7.5	35.4 (33.4–37.4)	517	36.8 (33.9–39.8)	688	34.3 (31.7–36.8)
Fair/poor	1,044	6.5	30.6 (28.6–32.6)	451	30.7 (27.7–33.7)	593	30.5 (28.0–33.1)
**Smoking status**							
Current	340	1.9	9.3 (8.1–10.5)	140	8.4 (6.7–10.1)	200	10.1 (8.4–11.7)
Former	1,264	7.4	35.8 (33.9–37.8)	598	40.7 (37.7–43.7)	666	32.0 (29.5–34.4)
Never	1,808	11.3	54.8 (52.8–56.9)	700	50.8 (47.7–54.0)	1,108	58.0 (55.3–60.7)
**No. of chronic comorbid conditions (beyond cancer diagnosis)^c^**							
0	803	5.5	25.8 (23.9–27.7)	305	22.4 (19.7–25.1)	498	28.6 (26.0–31.1)
1	945	5.6	26.6 (24.8–28.3)	369	24.4 (21.7–27.0)	576	28.3 (25.9–30.8)
2	812	4.8	22.6 (20.8–24.3)	361	24.6 (21.9–27.3)	451	20.9 (18.8–23.1)
≥3	926	5.3	25.1 (23.3–26.9)	443	28.7 (25.9–31.4)	483	22.2 (19.9–24.5)
**Disability^d^**							
Mobility disability	527	3.0	14.3 (12.9–15.8)	197	13.3 (11.2–15.4)	330	15.2 (13.2–17.2)
Other disability	185	1.2	5.5 (4.6–6.5)	86	6.1 (4.6–7.5)	99	5.1 (3.8–6.3)
No disability	2,774	16.9	80.1 (78.5–81.8)	1,195	80.7 (78.2–83.1)	1,579	79.7 (77.4–82.1)

Abbreviation: BMI, body mass index.

a Numbers are unweighted; percentages and 95% CIs are weighted; percentages may not add to 100 because of rounding.

b Eight respondents with missing data on age were excluded.

c The number of chronic comorbid conditions is based on affirmative responses to having ever been told by a doctor or health professional that respondent has the condition. Conditions included arthritis, chronic obstructive pulmonary disease, cirrhosis or hepatitis, dementia, diabetes, heart disease, hypertension, stroke, and weak or failing kidneys.

d Disability is based on responses of “a lot” or “cannot do at all” to the 6 disability types (mobility, vision, hearing, self-care, cognitive, communication). All respondents reporting a mobility disability were categorized as “mobility disability,” respondents reporting any other non–mobility disability were categorized “other disability,” and respondents reporting no disability were categorized as “no disability.”

Among cancer survivors, 33.9% (34.9% female, 32.7% male) were inactive, 28.1% (29.7% female, 26.2% male) were insufficiently active, and 38.0% (35.5% female, 41.2% male) were sufficiently active during leisure time in 2024 (Table 2). The prevalence of inactivity differed by age overall, with prevalence highest among cancer survivors aged 75 years or older (43.1%). Males were more likely than females (41.2% vs 35.5%) and non-Hispanic White adults (40.2%) were more likely than non-Hispanic Black (27.6%) or Hispanic adults (29.8%) to be sufficiently active. The prevalence of inactivity was higher among cancer survivors who reported having lower education levels (vs higher education levels), being widowed (vs being married/partnered or single/separated), living in rural areas (vs living in large central metropolitan areas), having obesity (vs being underweight/having a healthy weight or being overweight), and currently smoking (vs formerly or never smoking). Half of cancer survivors who described their health status as excellent or very good (57.8%) or reported no additional comorbid chronic conditions (51.7%) were sufficiently active. Generally, inactivity was more common among cancer survivors reporting poorer self-reported health status and more chronic diseases compared with those reporting better health factors. Cancer survivors who reported mobility disability were more likely to be inactive (64.6%) relative to both survivors who reported any other disability (45.9%) and those who reported no disability (27.7%).

**Table 2:** Unadjusted Prevalence of Leisure-Time Physical Activity Among Self-Reported Cancer Survivors (N = 3,365) by Demographic Characteristics and Health Status, National Health Interview Survey, 2024^a,b,c^

Characteristic	Total	Inactive		Insufficiently active		Sufficiently active		*P* value^d^
		No.	% (95% CI)	No.	% (95% CI)	No.	% (95% CI)	
Overall	3,365	1,143	33.9 (31.9–35.9)	919	28.1 (26.3–30.1)	1,303	38.0 (36.0–40.0)	NA
**Age group, y**								
18–39	127	22	19.2 (11.4–30.4)^e^	38	32.2 (22.5–43.6)	67	48.7 (38.4–59.0)^e^	<.001
40–64	932	273	29.4 (25.9–33.1)^f^	270	30.0 (26.6–33.7)	389	40.6 (36.9–44.4)^f^	
65–74	1,049	340	31.2 (27.8–34.9)^g^	274	27.6 (24.4–31.0)	435	41.2 (37.6–44.8)^g^	
≥75	1,250	504	43.1 (39.9–46.4)^e,f,g^	336	26.1 (23.3–29.0)	410	30.8 (27.9–33.9)^e,f,g^	
**Sex**								
Male	1,417	448	32.7 (29.7–35.8)	360	26.2 (23.4–29.2)	609	41.2 (38.0–44.4)^e^	.02
Female	1,948	695	34.9 (32.2–37.6)	559	29.7 (27.3–32.2)	694	35.5 (32.8–38.2)^e^	
**Race and ethnicity**								
Hispanic	220	86	40.1 (32.6–48.0)	65	30.1 (23.3–38.0)	69	29.8 (23.2–37.4)^e^	.02
Non-Hispanic Black	260	109	38.6 (31.3–46.4)	83	33.8 (26.6–41.9)	68	27.6 (21.5–34.6)^f^	
Non-Hispanic White	2,717	894	32.7 (30.6–34.9)	728	27.1 (25.1–29.2)	1,095	40.2 (37.9–42.4)^e,f^	
Non-Hispanic Other	168	54	33.8 (25.0–43.9)	43	29.6 (21.2–39.6)	71	36.6 (28.2–46.0)	
**Education level**								
Less than high school	263	137	52.2 (44.4–59.9)^e,f^	63	22.8 (17.2–29.5)	63	25.0 (18.8–32.5)^e,f^	<.001
High school or equivalent	854	390	45.5 (41.3–49.8)^g,h^	233	28.5 (24.7–32.7)	231	26.0 (22.6–29.7)^g,h^	
Some college	949	329	33.5 (29.9–37.2)^e,g,i^	275	29.0 (25.6–32.6)	345	37.5 (33.7–41.4)^e,g,i^	
College	1,295	285	21.4 (18.9–24.2)^f,h,i^	348	28.7 (25.9–31.6)	662	49.9 (46.7–53.1)^f,h,i^	
**Marital status**								
Married/partnered	1,674	487	29.9 (27.5–32.5)^e,f^	463	28.4 (26.0–31.0)	724	41.6 (39.0–44.3)^e,f^	<.001
Widowed	694	291	47.0 (42.3–51.7)^e,g^	187	25.6 (22.0–29.6)	216	27.4 (23.7–31.4)^e,g^	
Single/separated	957	350	37.3 (33.2–41.6)^f,g^	258	29.0 (25.1–33.1)	349	33.7 (30.1–37.6)^f,g^	
**Urban–rural classification**								
Large central metropolitan	806	235	29.2 (25.4–33.2)^e^	232	30.3 (26.5–34.5)	339	40.5 (36.6–44.5)	.009
Large fringe metropolitan	693	224	34.5 (30.5–38.8)	178	25.8 (22.0–29.9)	291	39.7 (35.6–43.9)	
Medium and small metropolitan	999	326	33.6 (30.0–37.4)	290	29.9 (26.6–33.4)	383	36.6 (33.1–40.2)	
Rural	867	358	41.2 (36.5–46.2)^e^	219	24.9 (21.3–28.9)	290	33.9 (29.5–38.5)	
**BMI category**								
Underweight/healthy weight (<25 kg/m^2^)	1,080	295	29.3 (25.9–32.9)^e^	261	24.7 (21.7–28.0)^e^	524	46.0 (42.3–49.7)^e^	<.001
Overweight (≥25 to <30 kg/m^2^)	1,156	370	32.3 (29.1–35.6)^f^	312	27.5 (24.5–30.8)	474	40.2 (36.9–43.6)^f^	
Obesity (≥30 kg/m^2^)	1,074	446	39.1 (35.7–42.5)^e,f^	334	32.2 (28.8–35.9)^e^	294	28.7 (25.4–32.2)^e,f^	
**Health status**								
Excellent/very good	1,196	207	16.2 (13.8–18.9)^e,f^	294	26.0 (23.0–29.2)	695	57.8 (54.3–61.3)^e,f^	<.001
Good	1,171	413	35.0 (31.7–38.5)^e,g^	369	30.3 (27.3–33.5)	389	34.6 (31.4–38.0)^e,g^	
Fair/poor	996	523	52.5 (48.6–56.3)^f,g^	256	28.0 (24.6–31.6)	217	19.5 (16.9–22.5)^f,g^	
**Smoking status**								
Current	332	161	49.1 (42.2–56.1)^e,f^	87	26.5 (20.7–33.3)	84	24.4 (19.2–30.4)^e,f^	<.001
Former	1,242	437	36.3 (33.1–39.6)^e,g^	336	27.1 (24.3–30.1)	469	36.6 (33.4–40.0)^e^	
Never	1,785	542	29.7 (27.1–32.4)^f,g^	495	29.2 (26.6–31.9)	748	41.1 (38.4–43.9)^f^	
**No. of chronic comorbid conditions^k^**								
0	779	146	20.1 (16.9–23.9)^e,f,g^	209	28.1 (24.5–32.0)	424	51.7 (47.7–55.8)^e,f,g^	<.001
1	913	262	27.9 (24.4–31.6)^e,h,i^	241	28.6 (25.0–32.6)	410	43.5 (39.6–47.4)^e,h,i^	
2	782	282	37.1 (33.2–41.1)^f,h,j^	234	30.3 (26.6–34.2)	266	32.6 (28.9–36.5)^f,h,j^	
≥3	891	453	51.6 (47.5–55.7)^g,i,j^	235	25.7 (22.3–29.4)	203	22.7 (19.6–26.1)^g,i,j^	
**Disability^l^**								
Mobility disability	502	318	64.6 (59.4–69.5)^e,f^	121	23.2 (18.8–28.2)	63	12.2 (9.3–15.9)^e,f^	<.001
Other disability	178	76	45.9 (36.5–55.7)^e,g^	53	26.6 (19.0–35.9)	49	27.4 (19.8–36.7)^e,g^	
No disability	2,685	749	27.7 (25.6–29.8)^f,g^	745	29.1 (27.1–31.3)	1,191	43.2 (40.9–45.5)^f,g^	

Abbreviation: BMI, body mass index; NA, not applicable.

^a^ Physical activity levels are defined as inactive (0 min/week of aerobic activity), insufficiently active (1–149 min/week), or sufficiently active (≥150 min/week, ie, meeting federal guidelines).

^b^ Numbers are unweighted; percentages and 95% CIs are weighted; percentages may not add to 100 because of rounding.

^c^ Respondents missing physical activity data (n = 121) were excluded from analysis.

^d^ Determined by χ^2^ test for association.

^e-j^ Within a characteristic and physical activity outcome, subgroup pairs with the same superscripted letters are significantly different (*P* < .05, determined by Bonferroni corrected pairwise *t* tests).

^k^ The number of chronic comorbid conditions is based on affirmative responses to having ever been told by a doctor or health professional that respondent has the condition. Conditions included arthritis, chronic obstructive pulmonary disease, cirrhosis or hepatitis, dementia, diabetes, heart disease, hypertension, stroke, and weak or failing kidneys.

^l^ Disability is based on responses of “a lot” or “cannot do at all” to the 6 disability types (mobility, vision, hearing, self-care, cognitive, communication). All respondents reporting a mobility disability were categorized as “mobility disability,” respondents reporting any other non–mobility disability were categorized “other disability,” and respondents reporting no disability were categorized as “no disability.”

Cancer survivors living in communities with walking supports were less likely to report being physically inactive and more likely to report being sufficiently active relative to cancer survivors living in communities without those walking supports (Figure 1). Differences for inactive and sufficiently active categories were significant for all 6 types of support (*P* < .001 for *t* tests). Physical inactivity was highest among cancer survivors who reported no access to walking routes (49.3%; 95% CI, 43.7%–55.0%) and no access to relaxing places (48.2%; 95% CI, 44.3%–52.1%) in their local walking environment.

**Figure 1:**
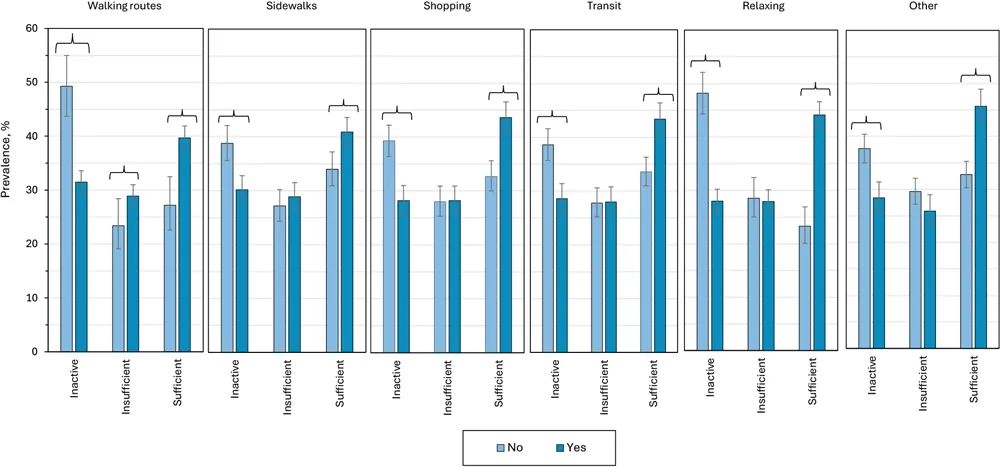
Unadjusted prevalence of leisure-time aerobic physical activity levels among self-reported cancer survivors by presence of community walking environment features that support walking in the local community. Physical activity levels are defined as inactive (0 minutes/week of aerobic activity), insufficiently active (1–149 minutes/week), or sufficiently active (≥150 minutes/week, ie, meeting federal guidelines). “Yes” indicates the presence of the support, and “No” indicates the absence of the support. Brackets indicate significant differences. Data source: National Health Interview Survey, 2024.

**Table:** 

Support	Unadjusted prevalence, %		
	Inactive	Insufficient	Sufficient
**Walking routes**			
No	49.3	23.4	27.2
Yes	31.5	28.9	39.7
**Sidewalks**			
No	38.8	27.2	34.0
Yes	30.2	28.9	40.9
**Shopping**			
No	39.3	28.0	32.7
Yes	28.2	28.2	43.6
**Transit**			
No	38.6	27.8	33.6
Yes	28.6	28.0	43.4
**Relaxing**			
No	48.2	28.5	23.3
Yes	28.0	27.9	44.1
**Other**			
No	37.7	29.6	32.8
Yes	28.4	25.9	45.7

Cancer survivors who reported crime as a barrier to walking safely in their community (10.5%; n = 351) were more likely to be physically inactive (44.6%; 95% CI, 38.3%–51.0%) and less likely to be sufficiently active (29.0%; 95% CI, 23.2%–35.5%) relative to cancer survivors reporting no crime (32.7% inactive; 95% CI, 30.6%–34.9% and 39.0% sufficiently active; 95% CI, 36.9%–41.2%) (Figure 2).

**Figure 2:**
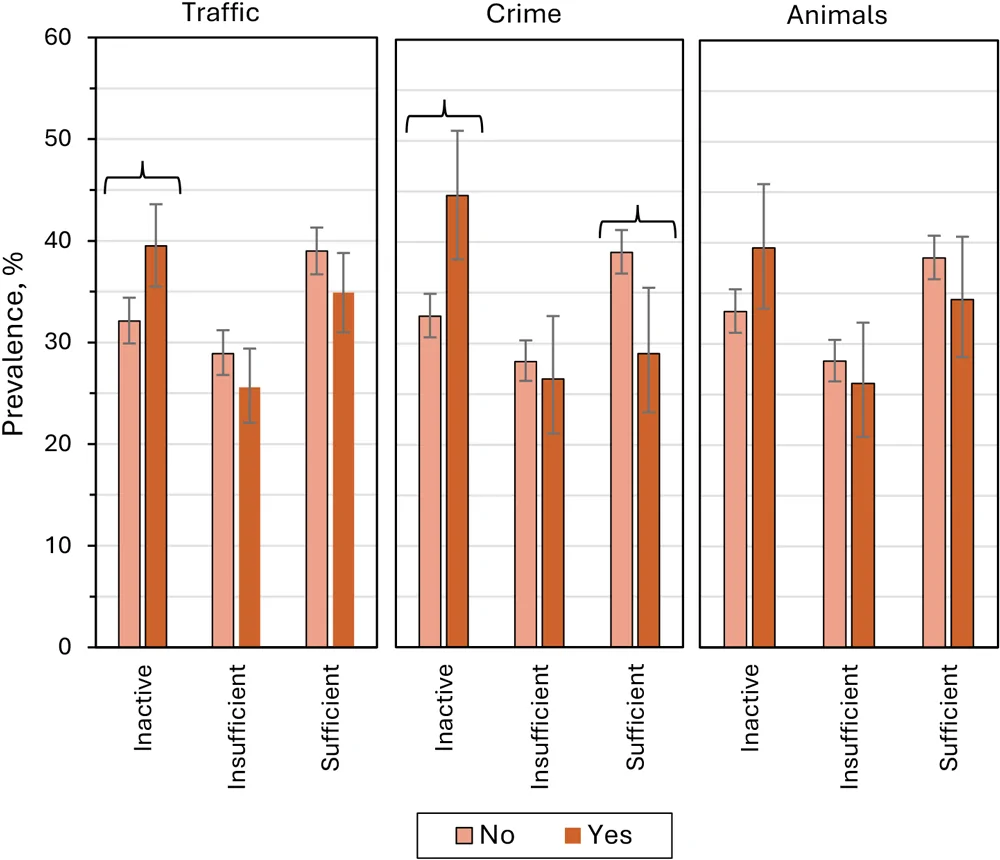
Unadjusted prevalence of leisure-time aerobic physical activity levels among self-reported cancer survivors by presence of community walking environment features that make it unsafe to walk (barriers) in the local community. Physical activity levels are defined as inactive (0 minutes/week of aerobic activity), insufficiently active (1–149 minutes/week), or sufficiently active (≥150 minutes/week, ie, meeting federal guidelines). “Yes” indicates the presence of the barrier, and “No” indicates the absence of the barrier. Brackets indicate significant differences. Data source: National Health Interview Survey, 2024.

**Table:** 

Barrier	Unadjusted prevalence, %		
	Inactive	Insufficient	Sufficient
**Traffic**			
No	32.1	28.9	39.0
Yes	39.5	25.6	34.9
**Crime**			
No	32.7	28.2	39.0
Yes	44.6	26.5	29.0
**Animals**			
No	33.2	28.3	38.5
Yes	39.5	26.1	34.4

Nearly half of inactive cancer survivors (49.5%; 95% CI, 45.1%–53.9%) reported that weather-related barriers to walking almost always made them less likely to walk, whereas among sufficiently active survivors, 23.0% (95% CI, 19.5–26.8%) reported that weather-related barriers to walking almost always made them less likely to walk (Figure 3).

**Figure 3:**
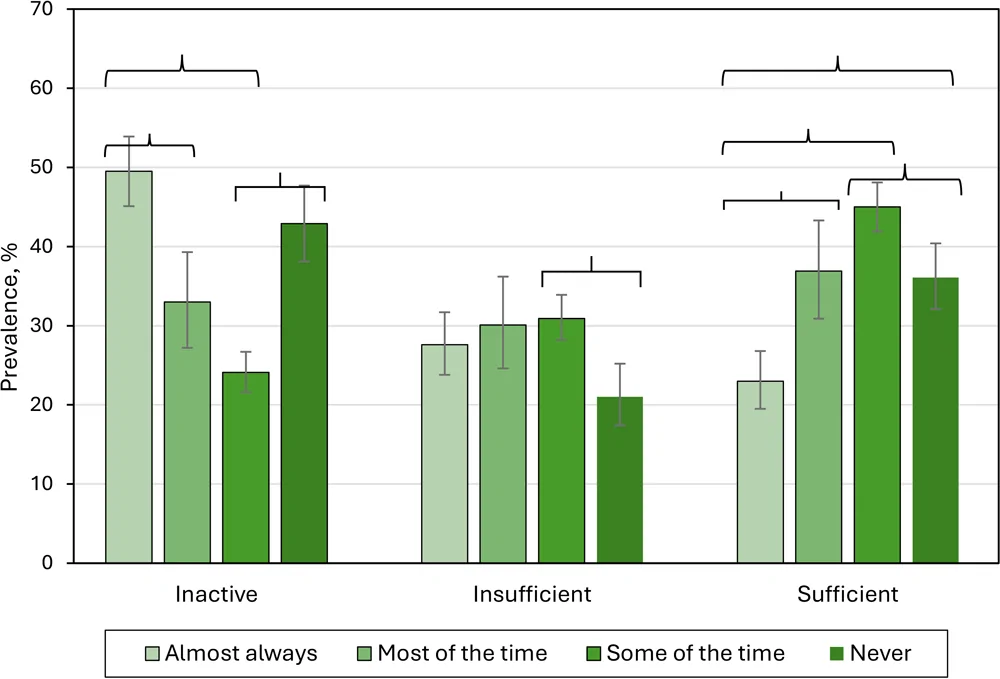
Unadjusted prevalence of leisure-time aerobic physical activity levels among self-reported cancer survivors by frequency of weather-related barriers to walking. Physical activity levels are defined as inactive (0 minutes/week of aerobic activity), insufficiently active (1–149 minutes/week), or sufficiently active (≥150 minutes/week, ie, meeting federal guidelines). The survey question asked, “How often does the weather make you less likely to walk?” Brackets indicate significant differences. Data source: National Health Interview Survey, 2024.

**Table:** 

Response	Unadjusted prevalence, %		
	Inactive	Insufficient	Sufficient
Almost always	49.5	27.6	23.0
Most of the time	33.0	30.1	36.9
Some of the time	24.1	30.9	45.0
Never	42.9	21.0	36.1

## Discussion

Our study indicates that among cancer survivors, roughly one-third each were inactive, insufficiently active, and sufficiently active in 2024, and that associations exist between physical activity and sociodemographic factors, health factors, and perceptions of the local walking environment. Overall, 62% of cancer survivors did not meet federal aerobic physical activity guidelines (were either inactive or insufficiently active). The prevalence of inactivity was higher among survivors with multiple chronic conditions or those reporting fair or poor health status, consistent with prior research (6,7,25,33). Cancer survivors reporting mobility disability were more likely to be inactive, as were those living in counties classified as rural compared with survivors living in large central metropolitan counties. These findings are also consistent with prior work highlighting mobility limitations and geographic disparities in built environment features as factors affecting regular movement (9). Interventions to increase activity and decrease sedentary lifestyles among cancer survivors, particularly for survivors with multiple chronic conditions (7), are an important health promotion objective because the benefits of physical activity are greatest among those who are least active.

The Community Preventive Services Task Force (Community Guide) recommends built environment approaches as one way to increase physical activity. Examples include combining transportation system interventions with land use and environmental design and combining park, trail, and greenway infrastructure interventions with community engagement strategies, such as structured physical activity programs and social interaction opportunities (18,34,35). Our study suggests that these approaches may also apply to cancer survivors because survivors living in communities with access to features that support walking had higher levels of physical activity compared with survivors without such access. Interventions designed to increase physical activity among cancer survivors, such as community engagement and programs promoting structured physical activity and social interaction, can be paired with efforts to increase access to built and natural environment features that promote physical activity, especially among survivors more likely to be inactive, such as survivors managing multiple chronic conditions (36). By improving access to safe public spaces designed for physical activity, relaxation, and social interaction, these combined interventions may increase the number of survivors engaging in physical activity. For example, walking groups among widowed survivors may promote physical activity along with social cohesion.

### Strengths and limitations

Study strengths include analysis of a large sample of US cancer survivors using nationally representative data. Limitations include self-reported data subject to social desirability and recall bias and a cross-sectional survey design that limits inferences of causality and directionality of observed associations. The study population included cancer survivors reporting mobility and other disabilities, limiting direct comparison with previous reports that excluded these groups. The analysis did not consider cancer type, severity of disease, or time since cancer diagnosis, each of which may affect access to and ability to engage in physical activity. Built environment features that influence walking in rural areas may be different from those in urban areas and may not have been fully captured in the survey. The aerobic physical activity outcome captures all leisure-time activities that elevate heart rate to moderate or vigorous levels regardless of activity type or location and may not reflect use of near-home built environment features. This study did not account for muscle-strengthening activity, which is recommended by the Physical Activity Guidelines for Americans and American Cancer Society Physical Activity Guideline for Cancer Survivors (10,12). Our study results were not adjusted for confounding, which was beyond the scope of this descriptive report; observed differences between groups should be interpreted with caution because they do not account for other factors that may influence the relationship between physical activity and a variable. Furthermore, the sample adult response rate for NHIS 2024 was 47.9%; however, base weights were adjusted for nonresponse (28).

### Conclusion

In 2024, most cancer survivors did not meet the federal guidelines for aerobic physical activity during leisure time, with 1 in 3 reporting inactivity. Among built environment features, we observed the largest differences in physical activity for survivors reporting access to relaxing destinations and walking routes within walking distance relative to those without access. Our findings support previous research that showed that environmental features supporting walking were positively associated with increased physical activity among cancer survivors (9) and expand this area of research by describing associations with various physical activity levels and examining associations with supports individually. Built environment features that encourage walking may increase physical activity among cancer survivors. To enhance opportunities for active transportation and leisure-time physical activity among cancer survivors, public health practitioners and other partners across a wide range of sectors may consider working together to implement and evaluate evidence-based strategies that include land use, environmental design, and community engagement.
